# Over-expression of hsa_circ_0088214 suppresses tumor progression by inhibiting Akt signaling pathway in osteosarcoma

**DOI:** 10.1186/s13018-023-03873-8

**Published:** 2023-05-26

**Authors:** Zhiwei Hao, Yiqun Yang, Daxia Xu, Hongyong Feng, Kunpeng Li, Changbin Ji, Man Li, Honglei Zhang

**Affiliations:** 1 Department of Orthopaedics, Liaocheng People’s Hospital, No 67 Dongchang West Road, Liaocheng City, 252000 Shandong Province People’s Republic of China; 2 Department of Cardiology, Liaocheng People’s Hospital, No 67 Dongchang West Road, Liaocheng City, 252000 Shandong Province People’s Republic of China

**Keywords:** Osteosarcoma, Circular RNA, Hsa_circ_0088214, Tumor progression, Akt

## Abstract

**Background:**

To explore the effect of has_circ_0088214 in osteosarcoma cells and corresponding mechanisms.

**Methods:**

Osteosarcoma cell line MG63 and U2OS were selected in this study. Wound-healing and matrigel transwell assays were performed to detect migration and invasion capacities. CCK-8 assay was used to measure cell growth and cisplatin resistance. Cell apoptosis was observed by Hoechst 33342 staining after H_2_O_2_ induce. Western Blot was used to detect protein expression level. The rescue experiments were also performed using an Akt activator SC79.

**Results:**

Hsa_circ_0088214 was down-regulated in osteosarcoma cells compared to normal osteoblast cells. Over-expression of has_circ_0088214 significantly reduced osteosarcoma cells invasion, migration and resistance to cisplatin, but the apoptotic ratio was increased. The phosphorylation level of Akt could be regulated by hsa_circ_0088214, and rescue experiments proved Akt signaling pathway took part in above biological processes.

**Conclusion:**

Up-regulation of hsa_circ_0088214 suppresses invasion, migration, cisplatin resistance but promoting apoptosis induced by H_2_O_2_ by inhibiting Akt signaling pathway in osteosarcoma.

## Introduction

Osteosarcoma is one of the most common malignant tumors in the world [1]. The patients who suffer from osteosarcoma are mostly children or adolescents under the age of 20. Osteosarcoma accounts for nearly half of tumors occurring in childhood period, and more than three-quarters of the patients are at the age between 15 and 25, with more male patients than female patients [2–4].

Osteosarcoma usually occurs in rapid growth periods of bone, it’s developed from osteoblasts and characterized in earlier onset age, higher metastasis and more rapid growth [5]. Most patients with osteosarcoma have metastasis at the time of diagnosis and the most common metastasis region are lung and other bones. The five-year survival rate of patients without metastasis is 60–70%, while it’s only about 20% for patients with metastasis [6]. Therefore, it is urgently needed to clarify the etiology and explore an effective systemic treatment for osteosarcoma.

In recent years, it has been found that non-coding RNAs (ncRNAs) act as a class of molecules that play an important role in the regulation of gene expression. It will be helpful to explore the molecular mechanism of tumor occurrence and development, and find new therapeutic targets [7, 8]. Circular RNAs (circRNAs) were first discovered in the 1990s. They are a special kind of ncRNAs formed end to end in the process of transcription and highly conserved in the evolutionary process. In previous researches, circRNAs have been found to play an important role in many physiological and pathological processes, and their role in the occurrence and development of malignant tumors has attracted more attention [9–11]. Compared to general linear RNAs, circRNAs are more stable and more difficult to be degraded, and it can resist the digestion of RNA enzymes. These findings suggest that circRNAs may be important molecules in regulating gene expression.

At present, the role of circRNAs in liver cancer, stomach cancer, breast cancer, colon cancer, lung cancer and other cancers has attracted widespread attention [12–18]. Studies have found that circRNAs mainly regulate tumor proliferation, apoptosis, invasion and migration. Due to their stable nature, some circRNAs closely related to tumorigenesis are expected to become specific markers. In this study, we aimed to explore the important effect of circRNAs, hsa_circ_0088214 particularly, in regulating osteosarcoma progression.

## Materials and methods

### Cell culture

Human osteosarcoma cells, MG63 and U2OS were obtained from the American Type Culture Collection (ATCC, USA). All the cell lines were maintained in Dulbecco’s modified Eagle’s medium (DMEM) supplemented with 10% fetal bovine serum (Gibco, USA). All the cells were cultured in an incubator at 37 °C with 5% CO_2_.

### Cell counting kit-8 assay

Three thousand cells were seeded into 96-well plates and cultured overnight. Absorbance at 450 nm (OD450) was recorded at the 1 day, 2 day, 3 day, 4 day and 5 day time points respectively according to the instructions of the Cell counting kit-8 (CCK-8 kit; Beyotime, China). For drug resistance assay, 15,000 cells were treated with different concentration of cisplatin for 24 h. Finally, viability curves were plotted.

### Wound healing assay

Osteosarcoma cells were cultured in 6-well plates, and cell monolayer was subsequently scratched with a 200 µL pipette tip. Representative images of cell migration were captured 0 and 24 h after scratching.

### Invasion assay

Matrigel was dissolved overnight at 4 °C, and diluted with DMEM. 40 μL diluted matrigel was added into the upper chamber and solidified after incubation at 37 °C for 2 h. 20,000 cells with 200 μL DMEM was added into the upper chamber, and 600 μL complete medium was added into the lower chamber. Non-invaded cells were wiped with cotton swabs after culturing for 24 h, and the left cells were fixed with 4% paraformaldehyde for 15 min, stained with 1% crystal violet for 5 min. Images of cell invasion were captured under a light microscope.

### qRT-PCR

RNA extraction and quantitative real-time PCR (qRT-PCR) were performed according to the manufacturer’s instructions. The qRT-PCR was conducted using Revert Aid First Strand cDNA Synthesis Kit (Thermo Fisher Scientific, US) and the Maxima SYBR Green qPCR Master Mix (Thermo Fisher Scientific). Relative gene expression was calculated by the comparative CT method (ΔΔCT), and GAPDH was used as a housekeeping gene.

### Western blot

Cells were lysed with RIPA buffer with 1% proteinase and phosphatases inhibitors. The proteins were separated by SDS-PAGE gel electrophoresis and transferred to PVDF membranes. The membranes were blocked with 5% BSA and incubated with primary antibody overnight at 4 °C. The primary antibodies were used as follows: anti-E-cadherin (1:1000; Cell Signaling Technology, USA), anti-Snail (1:1000; Cell Signaling Technology, USA), anti-Bax (1:1000; Cell Signaling Technology, USA), anti-P-Akt (1:1000; Cell Signaling Technology, USA), anti-Akt (1:1000; Cell Signaling Technology, USA) and anti-β-actin (1:1000; Cell Signaling Technology, USA). Then, the membrane was washed with TBST for three times and incubated with the second antibody for 1 h at room temperature. After washing with TBST for three times, the expression of targeted protein was analyzed by using the electrochemiluminescence reagent.

### Hoechst 33342 staining

Cells were seed into 6-well plate and cultured to more than 80% confluence. Then cells were treated with 600 μM H_2_O_2_ for 12 h to induce apoptosis. After Hoechst 33342 staining, the nucleus of normal cells is ordinarily blue, whereas the nucleus of apoptotic cells will be densely stained, or clumpy densely stained, with a little whitish.

### Cell cycle detection

Cells were seed in 6-well plate and treated with vector or circular RNA overexpression plasmid. Propidium iodide (PI) with the concentration of 20 μg/mL was used for DNA staining and cells were detected by flowcytometry and the data was analyzed by Flowjo software.

### Apoptosis analysis

Cells were seed in 6-well plate and treated with vector or circular RNA overexpression plasmid. Cells were treated with 600 μM H_2_O_2_ for 12 h in order to induce cell apoptosis and stained with Annexin V/PI. Cell apoptosis ratio was detected by flowcytometry.

### Bioinformatic analysis

In order to explore the downstream proteins regulated by hsa_circ_0088214, CircInteractome database (https://circinteractome.nia.nih.gov/) was used for predicting. And the classic protein interaction database STRING (https://cn.string-db.org/) was used to show the interaction of downstream proteins.

### Statistical analysis

All data were presented as the mean ± standard deviation (SD). The differences among three groups or more were analyzed by one-way analysis of variance (one-way ANOVA). *P* < 0.05 was considered to be statistically significant.

## Results

### Hsa_circ_0088214 was down-regulated in osteosarcoma cells compare to normal osteoblast

**Fig. 1:**
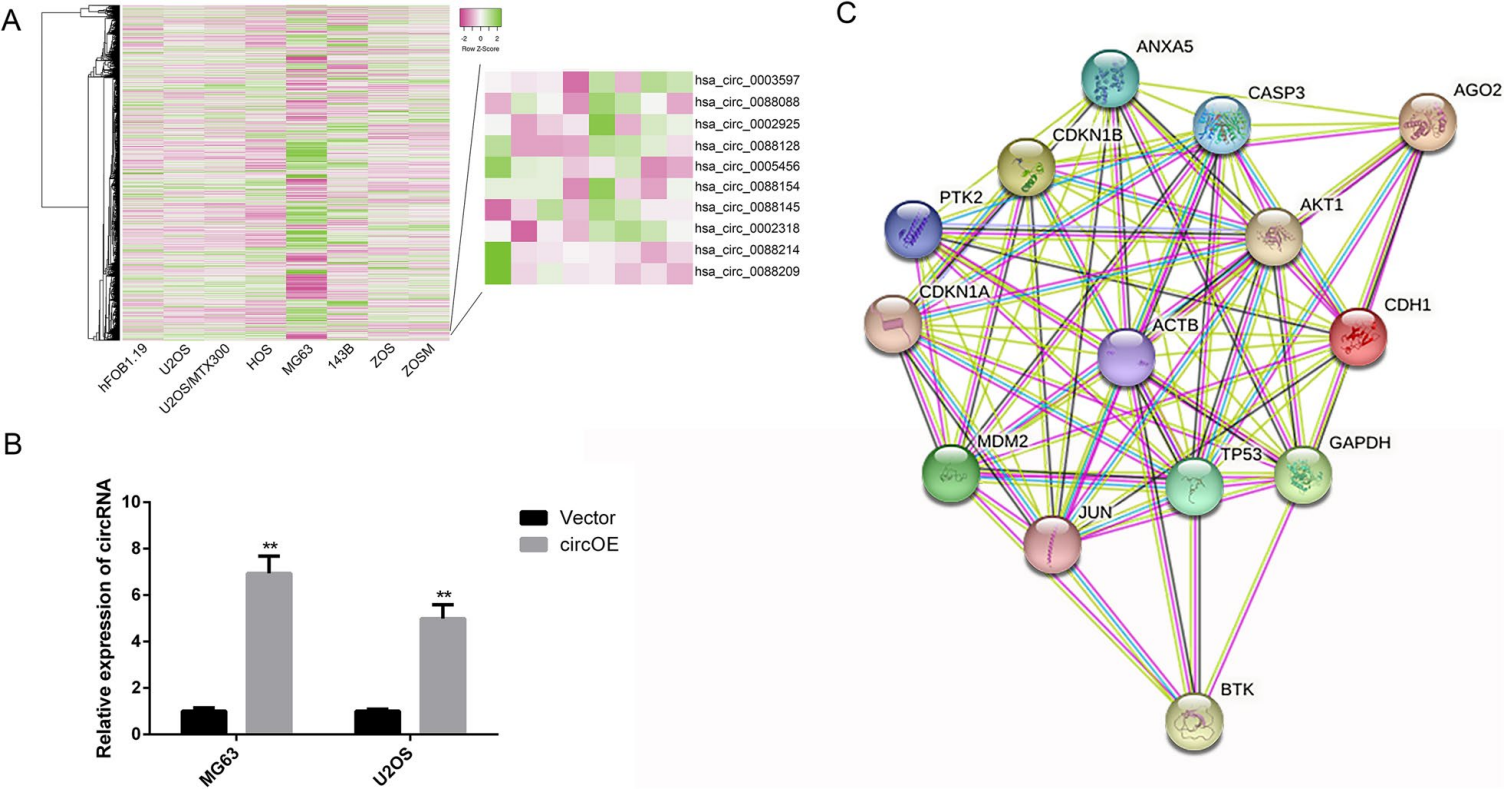
Hsa_circ_0088214 was low-expressed in osteosarcoma cells. **A** Heat map was drawn according to GEO data GSE96964. **B** Relative expression of hsa_circ_0088214 after tranfecting with over-expression or blank plasmids. **C** Network of protein–protein interaction. ^**^P < 0.01 compared to Vector group

First, GSE96964 microarray expression dataset was downloaded from the GEO database. After comparing circRNAs in hFOB1.19 cells with those in osteosarcoma cells, hsa_circ_0088214 was discovered to be down-regulated in a series of osteosarcoma cells than in normal osteoblast cell line hFOB1.19 (Fig. 1A). In order to up-regulate the expression of hsa_circ_0088214, over-expression plasmid of hsa_circ_0088214 was transfected into osteosarcoma cells MG63 and U2OS, and the effect was verified by q-RT PCR (Fig. 1B). In order to explore the regulation mechanism, we used CircInteractome database to predict RNA-binding proteins and AGO2 protein has the binding sites with hsa_circ_0088214. As shown in the results of STRING database, AGO2 could interact with AKT1 directly, which is proved by experimental verification (Fig. 1C). So we guess hsa_circ_0088214 takes part in osteosarcoma progression via Akt signaling pathway.

### Over-expression of hsa_circ_0088214 inhibited the invasion and migration of osteosarcoma cells by suppress Akt signaling pathway

**Fig. 2:**
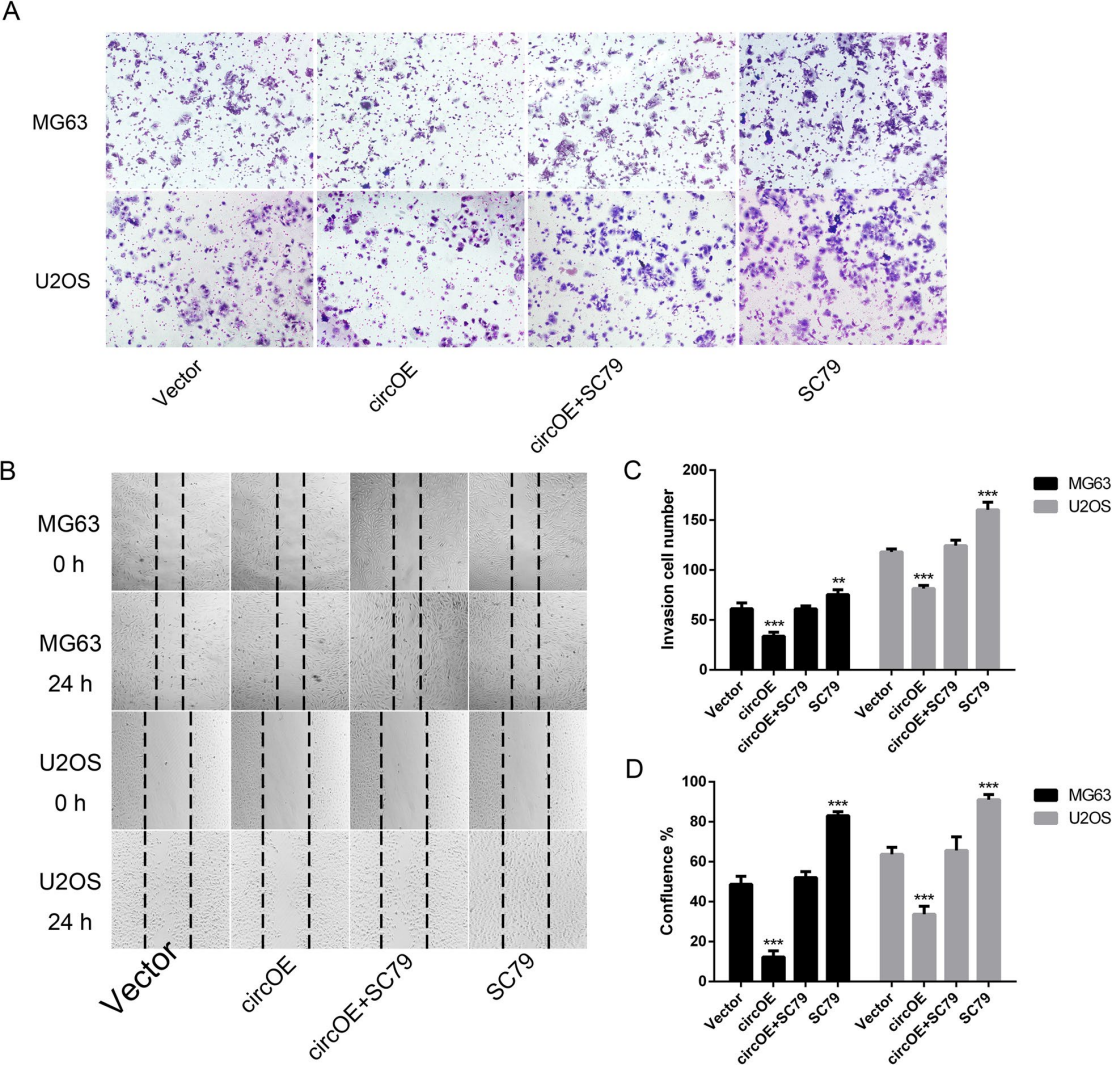
Over-expression of hsa_circ_0088214 suppressed osteosarcoma cells MG63 and U2OS invasion and migration. **A** Osteosarcoma cells invasion capacity treated with vector plasmid, circRNA over-expression plasmid, circRNA over-expression plasmid + SC79 or SC79. **B** Osteosarcoma cells migration capacity with above treatments. **C** Statistics for invasion capacity. ^**^P < 0.01, ^***^P < 0.001 compared to Vector group. **D** Statistics for migration capacity. ^***^P < 0.001 compared to Vector group

**Fig. 3:**
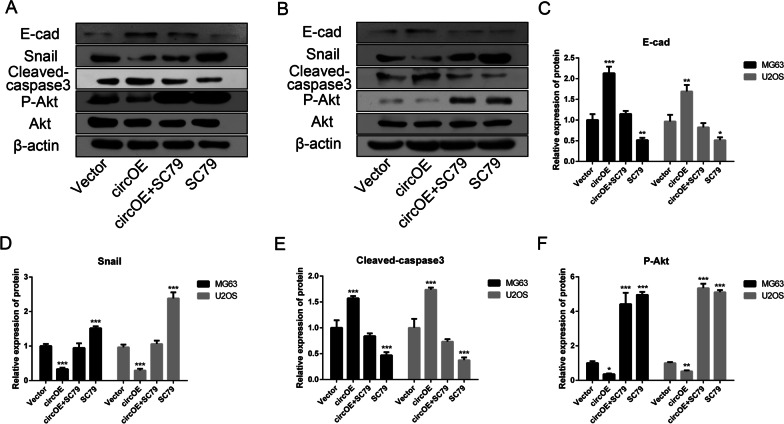
The protein expression levels of E-cadherin, Snail, cleaved-caspase3, P-Akt, Akt and β-actin detected by Western blot. **A** The protein expression levels in MG63 treated with vector plasmid, circRNA over-expression plasmid, circRNA over-expression plasmid + SC79 or SC79. **B** The protein expression levels in U2OS with above treatments. **C** Statistics for E-cadherin. ^*^P < 0.05, ^**^P < 0.01, ^***^P < 0.001 compared to Vector group. **D** Statistics for Snail. ^***^P < 0.001 compared to Vector group. **E** Statistics for cleaved-caspase3. ^***^P < 0.001 compared to Vector group. **F** Statistics for P-Akt. ^*^P < 0.05, ^**^P < 0.01, ^***^P < 0.001 compared to Vector group

To identify the potential role of hsa_circ_0088214 in osteosarcoma cells, invasion and migration capacities were detected by matrigel transwell assay (Fig. 2A, C) and wound-healing assay (Fig. 2B, D). It could be found that over-expression of hsa_circ_0088214 decreased the number of invasion ad migration cells, but the capacities of invasion and migration could be rescued after treating with Akt activator SC79. So it can be concluded that over-expression of hsa_circ_0088214 might suppress osteosarcoma cells MG63 and U2OS by inhibiting Akt signaling, and it can also be proved by the result of Western Blot (Fig. 3A, B). Up-regulation of hsa_circ_0088214 would decrease the phosphorylation level of P-Akt (Fig. 3F) and affect the expression of E-cadherin (Fig. 3C) and Snail (Fig. 3D) protein, which could be rescued by SC79.

### Hsa_circ_0088214 suppressed the proliferation and resistance to cisplatin but it promoted cell apoptosis in osteosarcoma cells

**Fig. 4:**
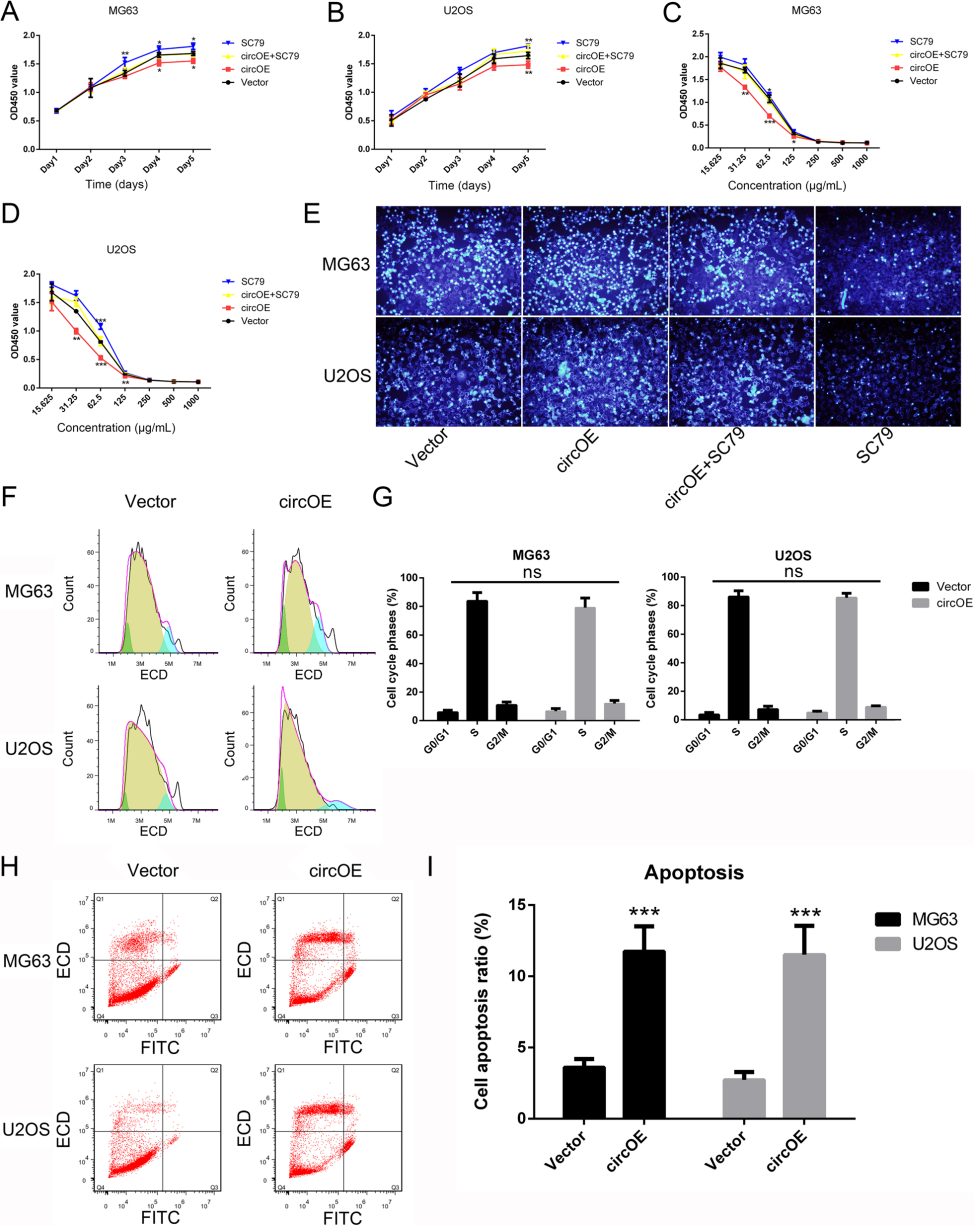
Over-expression of hsa_circ_0088214 suppressed osteosarcoma cells MG63 and U2OS growth, cisplatin resistance but increased apoptotic ratio induced by H_2_O_2_. **A** Growth curve of MG63 treated with vector plasmid, circRNA over-expression plasmid, circRNA over-expression plasmid + SC79 or SC79. ^*^P < 0.05, ^**^P < 0.01 compared to Vector group. **B** Growth curve of U2OS with above treatments. ^**^P < 0.01 compared to Vector group. **C** Drug resistance assay of MG63 with above treatments under different concentrations of cisplatin. ^*^P < 0.05, ^**^P < 0.01, ^***^P < 0.001 compared to Vector group. **D** Drug resistance assay of U2OS with above treatments under different concentrations of cisplatin. ^**^P < 0.01, ^***^P < 0.001 compared to Vector group. **E** Hoechst 33342 staining of MG63 and U2OS with above treatments induced by 600 μM H_2_O_2_ for 12 h. **F**, **G** Cell cycle analysis of MG63 and U2OS treated with vector plasmid or circRNA over-expression plasmid. **H**, **I** Apoptosis analysis of MG63 and U2OS treated with vector plasmid or circRNA over-expression plasmid induced by 600 μM H_2_O_2_ for 12 h. ^***^P < 0.001 compared to Vector group

The results of CCK-8 assay (Fig. 4A–D) revealed that over-expression of hsa_circ_0088214 could slow osteosarcoma cells growth and weaken their resistance to cisplatin obviously. These effects could also be blocked by Akt activator SC79. For cisplatin resistance detection, it could be found that IC50 concentration for hsa_circ_0088214 over-expressed cells was about 40 µg/mL, while for wild type cells was about 80 µg/mL (MG63) or 60 µg/mL (U2OS). Up-regulation of hsa_circ_0088214 could decrease cisplatin IC50 in osteosarcoma cells.

### Hsa_circ_0088214 facilitated osteosarcoma cells apoptosis induced by H_2_O_2_

After treating with H_2_O_2_ for 12 h, osteosarcoma cells were stained with Hoechst 33342 to show their nuclei. Figure 4E showed that cells with hsa_circ_0088214 had the highest apoptosis ratio and the worst cell shape, and SC79 treatment could decrease apoptosis ratio. The result of Western Blot (Fig. 3E, F) proved that the influence of hsa_circ_0088214 on cell apoptosis was mediated by Akt signaling pathway. Cell cycle and apoptosis detection were also conducted, and the results showed that hsa_circ_0088214 had little influence on cell cycles (Fig. 4F, G), but it could promote cell apoptosis induced by H_2_O_2_ (Fig. 4H, I), which also proved above conclusions.

## Discussion

As a new member of the non-coding RNAs, circRNAs have gained extensive attention. Series of studies have reported that circRNAs can be involved in a variety of diseases, in tumors particularly. CircRNAs can activate oncogene or inactivate tumor suppressor genes to take part in regulation of invasion, migration, proliferation, apoptosis, drug resistance and metastasis of tumors. However, the detailed functions and mechanisms of many circRNAs still remain unclear, and the regulatory effects of lots of circRNAs in osteosarcoma also need to be explored. In this study, we focused on the role of hsa_circ_0088214 in osteosarcoma, and clarifying the corresponding mechanisms.

It’s demonstrated that hsa_circ_0088214 is lower expression in osteosarcoma cells than normal osteoblast cell, and it could take part in regulation of invasion, migration, apoptosis and drug resistance in osteosarcoma cells. Up-regulation of hsa_circ_0088214 could inhibit metastasis and drug resistance capacities of osteosarcoma cells obviously, and the apoptotic cell number is increased inducing by H_2_O_2_. The phosphorylation level of P-Akt is also down-regulated in reaction to hsa_circ_0088214, and activation of P-Akt using SC79 can rescue invasion, migration, apoptosis and drug resistance capacities inhibited by hsa_circ_0088214. So it can be concluded that low-expression of hsa_circ_0088214 in osteosarcoma cells promotes cell invasion, migration and cisplatin resistance but suppresses apoptosis by regulating Akt signaling pathway.

It’s first demonstrated by experiments that over-expression of hsa_circ_0088214 could suppress progression of osteosarcoma cells and decrease their cisplatin resistance. Our findings raised the possibility to use hsa_circ_0088214 as biomarker for predicting biological behavior and cisplatin sensitivity in osteosarcoma. And these findings will helpful to facilitate the implementation of the precision medicine by determining an optimal therapy for each individual osteosarcoma patient.

## Data Availability

All data generated or analyzed during this study are available from the corresponding author upon reasonable request.
